# Lethal Injection Is Not Humane

**DOI:** 10.1371/journal.pmed.0040171

**Published:** 2007-04-24

**Authors:** The PLoS Medicine Editors

## Abstract

This month's article by Koniaris and colleagues provides further evidence that lethal injection is far from humane. The*PLoS Medicine* editors call on the US to abandon the death penalty.

This month's issue of *PLoS Medicine* contains a research article on three protocols used in lethal injection, the current method of execution for most US states. Despite the British Royal Commission on Capital Punishment advising against lethal injection half a century ago [1], the United Nations General Assembly affirming the desirability of abolishing the death penalty in 1971, and the European Union explicitly banning the death penalty in all circumstances [2], execution—predominantly by lethal injection—is still practiced in many countries. During 2005 at least 2,148 people were executed in 22 countries in cases recorded by Amnesty International; the actual numbers were certainly higher. The majority of these executions took place in China, where fleets of mobile execution vans have been deployed to facilitate prompt, low-profile executions by lethal injection. Iran, Saudi Arabia, and the US together with China accounted for 94% of executions in 2005 [3].

Following its introduction to the US in 1982, lethal injection became the primary method of execution there, largely replacing execution by hanging, firing squad, gas chamber, and electrocution. Each of these older methods has come to be seen as inhumane or excessively violent by most states, but each remains an option in a handful of others. Of the 53 executions in the US in 2006, all but one (an electrocution) were carried out using lethal injection [4].

In recent months, concerns over botched lethal injections have put the method on hold in a dozen or so of the 36 US states that have the death penalty. Following a particularly agonizing execution in December 2006, the US District Court ruled that California's lethal injection protocol was unconstitutional. The governors of Florida and Tennessee suspended executions pending review of their states' lethal injection protocols. A court ruling in December 2006 suspended Maryland's executions, and New Jersey is considering an outright ban on its death penalty following a 2004 court order requiring the state to justify its lethal injection process. Executions are on hold in several other states pending legal proceedings [4].

In this context, the editors of *PLoS Medicine* believe it is timely to publish a research article reporting shortcomings of lethal injection protocols. Strictly speaking, this article has little to do with medicine. Execution by lethal injection, even if it uses tools of intensive care such as intravenous tubing and beeping heart monitors, has the same relationship to medicine that an executioner's axe has to surgery. Nonetheless, there is a need for greater openness in public discussion and consideration of the death penalty, including its unpalatable details.

Challenges to the constitutionality of lethal injection have thus far been based largely on accounts of suffering resulting from unskilled administration. The American Medical Association, the American Nurses Association, the Society of Correctional Physicians, and a number of state medical boards have banned as unethical any causative role for medical professionals in executions [5]. Accordingly, in lethal injection procedures intravenous access has often been attempted (with frequent failures) by untrained staff, the execution mixture has precipitated and blocked IV tubes, and lethal doses have been unreliably calculated [6]. Anesthesia has failed, chemical burns have occurred, and suffering has proceeded for 30 minutes or longer. Although this suffering might be seen as a consequence of professional refusal to participate in executions, this refusal also appears to be one of the primary forces motivating reexamination of lethal injection by the courts.

The current article by Koniaris and colleagues gives further cause for concern by questioning whether, even if “perfectly” administered, the protocols would achieve their stated aim of causing death without inflicting inhumane punishment. The authors analyzed several cases from three states: California, North Carolina, and Virginia. (Texas, the state with the largest number of lethal injections, does not release data from executions.) These lethal injection protocols use the barbiturate thiopental (intended to sedate and to suppress breathing), the neuromuscular blocker pancuronium (which paralyzes, causing respiratory arrest but also preventing agonal movements that might indicate suffering), and the electrolyte potassium (intended to cause cardiac arrest). Such protocols are intended to provide redundancy, such that each drug is given at a dose that would by itself cause death. However, in analyzing data from actual executions, Koniaris and colleagues report that thiopental and potassium do not consistently result in death. In fact, individuals undergoing execution have continued to breathe after the injection of thiopental, and their hearts have continued to beat following injection of potassium; in these cases, the authors conclude, it is quite likely that those being executed have experienced asphyxiation while conscious and unable to move, and possibly an intense burning pain throughout the body from the potassium injection.

Each of the editors of *PLoS Medicine* opposes the death penalty. It is not our intention to encourage further research to “improve” lethal injection protocols. As editors of a medical journal, we must ensure that research is ethical, and there is no ethical way to establish the humaneness of procedures for killing people who do not wish to die. Human research to further the ends of governments at the expense of individual lives is an obvious violation of the Declaration of Helsinki, which was conceived largely in response to the atrocities of Nazi “medicine” in order to articulate an international standard for ethical human experimentation [7]. Whatever local law might say in a given place and time, no ethical researcher would propose a study to establish such procedures, no ethical reviewers would approve it, and no ethical journal would publish it. The acceptability of lethal injection under the US Constitution's Eighth Amendment ban on inhumane punishment has never been established; the data presented by Koniaris and colleagues adds to the evidence that lethal injection is simply the latest in a long line of execution methods that have been found to be inhumane. It is time for the US to join the majority of countries worldwide in recognizing that there is no humane way of forcibly killing someone.

Apart from the issue of whether humane execution can exist, we must also consider the accuracy of convictions resulting in death sentences. Execution of wrongfully sentenced individuals is obviously unacceptable, yet between 1973 and 2004 in the US, 118 prisoners who had been sentenced to death were later released on grounds of innocence [8]. Of 197 convictions in the US that were subsequently exonerated by DNA evidence, 14 were at one time sentenced to death or served time on death row [9]. Racial bias in sentencing likely accounts for much of this error; more than half of the exonerees were African Americans, and the rate of death sentences in the US among those convicted of killing a white victim is considerably higher than for murderers of blacks. Given this potential for fatal error, how can any objective person support the death penalty, which allows for no correction?

We support the recent decision of Craig Watkins, the new district attorney of Dallas, Texas, to examine hundreds of cases over the past 30 years to see whether DNA tests might reveal wrongful convictions [10]. Such errors are inevitable when an implicit goal of sentencing, and particularly of imposing the death penalty, is not rational but emotional: the desire for revenge. As one law professor stated in a recent *New York Times Magazine* article on lethal injection, “Retribution, the conscious affliction of pain and suffering because and only because some people deserve it, is the essence of punishment” [11]. But if the personal satisfaction of seeing criminals “get what they deserve” really reflects the intentions of Americans, why has the US seen a transition away from firing squads, hanging, or even drawing and quartering? Why was capital punishment illegal for a decade until it was reinstated by a Supreme Court ruling in 1976? Why have some US states rejected the death penalty completely, and others suspended its use? Why has the US followed the course associated with totalitarian states and rejected by other democracies in this matter?

Clearly, the death penalty is a matter of profound ambivalence in American society. Courts and state governments are saying that if capital punishment exists, it must not be cruel or visibly violent. Physicians and nurses are saying that their involvement in executions is below any acceptable conception of professional ethics. How to reconcile the needs of a society given to vengeance but outwardly abhorrent of cruelty or violence, trusting of medical science's trappings but indifferent to their use in killing, expecting the highest ethics of its physicians but willing to medicalize the execution chamber? The new data in *PLoS Medicine* will further strengthen the constitutional case for the abandonment of execution in the US. As a moral society, the US should take a leading role in the abandonment of executions worldwide.
